# The role of the clinician in the multi-omics era: are you ready?

**DOI:** 10.1007/s10545-017-0128-1

**Published:** 2018-01-23

**Authors:** Clara D. M. van Karnebeek, Saskia B. Wortmann, Maja Tarailo-Graovac, Mirjam Langeveld, Carlos R. Ferreira, Jiddeke M. van de Kamp, Carla E. Hollak, Wyeth W. Wasserman, Hans R. Waterham, Ron A. Wevers, Tobias B. Haack, Ronald J.A. Wanders, Kym M. Boycott

**Affiliations:** 1 Department of Pediatrics and Clinical Genetics, Academic Medical Centre, Amsterdam, The Netherlands; 2 Departments of Pediatrics, Centre for Molecular Medicine and Therapeutics, BC Children’s Research Institute, University of British Columbia, Vancouver, BC Canada; 3 Deparment of Pediatrics (Room H7-224), Emma Children’s Hospital, Academic Medical Centre, Meibergdreef 9, 1105 AZ Amsterdam, The Netherlands; 4 Department of Pediatrics, Salzburger Landeskliniken (SALK) and Paracelsus Medical University (PMU), Salzburg, Austria; 5 Institute of Human Genetics, Helmholtz Zentrum München, Neuherberg, Germany; 6 Institute of Human Genetics, Technische Universität München, Munich, Germany; 7 Departments of Medical Genetics, Centre for Molecular Medicine and Therapeutics, BC Children’s Research Institute, Vancouver, BC Canada; 8 Departments of Biochemistry, Molecular Biology, and Medical Genetics, Cumming School of Medicine, University of Calgary, Calgary, CA Canada; 9 Alberta Children’s Hospital Research Institute, University of Calgary, Calgary, CA Canada; 10 Department of Endocrinology and Metabolism, Academic Medical Centre, Amsterdam, The Netherlands; 11 National Human Genome Research Institute, National Institutes of Health, Bethesda, MD USA; 12 Department of Clinical Genetics, VU University Medical Center, Amsterdam, The Netherlands; 13 Laboratory Genetic Metabolic Diseases, Department of Clinical Chemistry, Laboratory Division & Department of Pediatrics, Academic Medical Centre, Amsterdam, The Netherlands; 14 Department of Laboratory Medicine, Translational Metabolic Laboratory, Radboud University Medical Centre, Nijmegen, The Netherlands; 15 Institute of Medical Genetics and Applied Genomics, University of Tuebingen, Tuebingen, Germany; 16 Children’s Hospital of Eastern Ontario Research Institute, University of Ottawa, Ottawa, Canada

**Keywords:** Inherited metabolic disease, Genomics, Metabolomics, Diagnosis, Treatment, Precision medicine

## Abstract

Since Garrod’s first description of alkaptonuria in 1902, and newborn screening for phenylketonuria introduced in the 1960s, P4 medicine (preventive, predictive, personalized, and participatory) has been a reality for the clinician serving patients with inherited metabolic diseases. The era of high-throughput technologies promises to accelerate its scale dramatically. Genomics, transcriptomics, epigenomics, proteomics, glycomics, metabolomics, and lipidomics offer an amazing opportunity for holistic investigation and contextual pathophysiologic understanding of inherited metabolic diseases for precise diagnosis and tailored treatment. While each of the -omics technologies is important to systems biology, some are more mature than others. Exome sequencing is emerging as a reimbursed test in clinics around the world, and untargeted metabolomics has the potential to serve as a single biochemical testing platform. The challenge lies in the integration and cautious interpretation of these big data, with translation into clinically meaningful information and/or action for our patients. A daunting but exciting task for the clinician; we provide clinical cases to illustrate the importance of his/her role as the connector between physicians, laboratory experts and researchers in the basic, computer, and clinical sciences. Open collaborations, data sharing, functional assays, and model organisms play a key role in the validation of -omics discoveries. Having all the right expertise at the table when discussing the diagnostic approach and individualized management plan according to the information yielded by -omics investigations (e.g., actionable mutations, novel therapeutic interventions), is the stepping stone of P4 medicine. Patient participation and the adjustment of the medical team’s plan to his/her and the family’s wishes most certainly is the capstone. Are you ready?

## Introduction

**Fig. 1:**
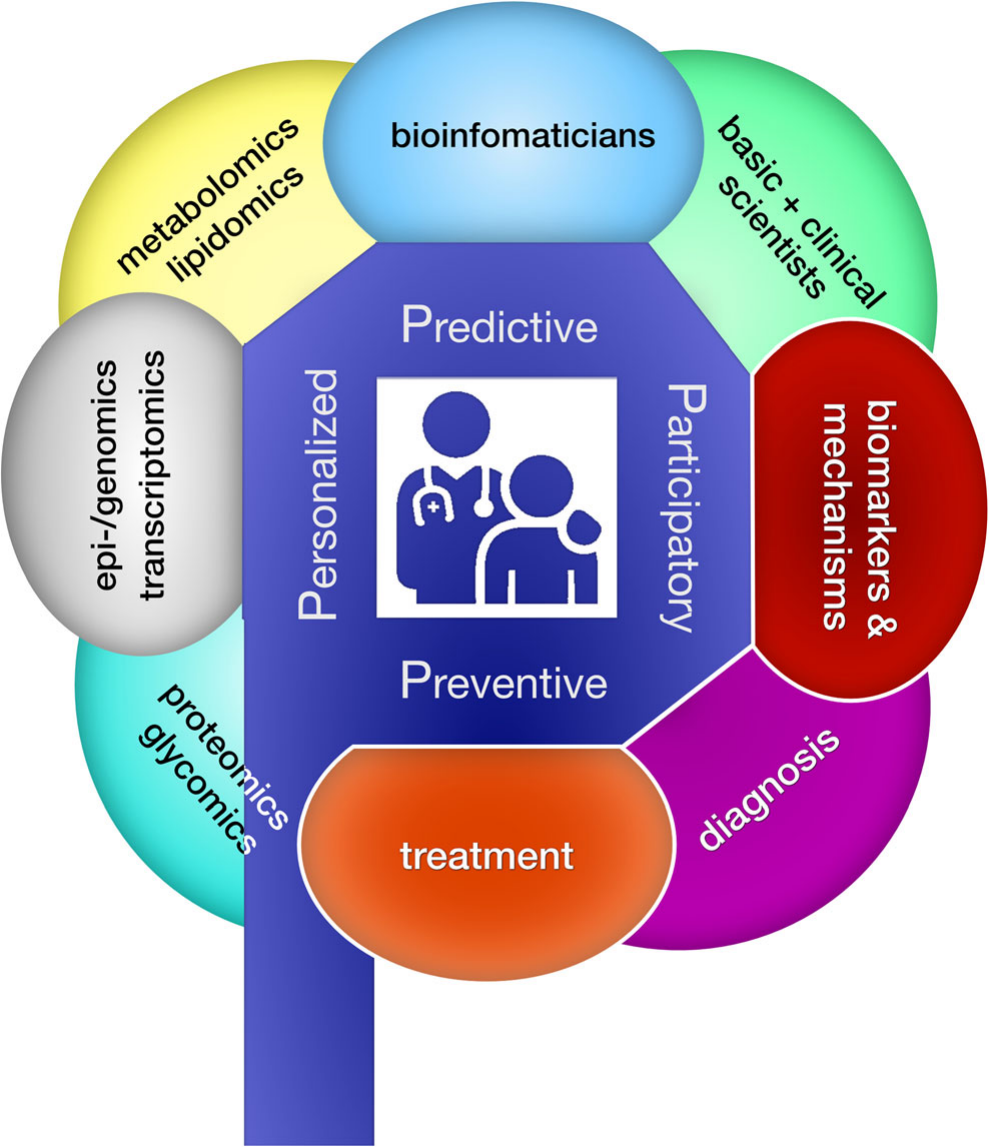
The clinician as connector in the omics-era: making true the 4Ps of precision medicine

For the clinician in the multi-omics era, the responsibilities defined by Hippocrates remain the same—that is to characterize, diagnose, manage and, where possible, treat his or her patients to the best of his/her capabilities and that of available modern technology (Karagiannis 2014). Revolutionary advances in the latter, catalyzed by the Human Genome Project in the beginning of the twenty-first century and fueled since by academia and biotechnology companies, now enable a holistic characterization of our patients using multi-omic approaches (Goodwin et al 2016). Inherited metabolic diseases (IMDs)—characterized by defects in a metabolic pathway or process resulting in a deficiency of energy and building blocks and often an accumulation of (toxic) metabolites—is an exciting field within the -omics era. It also represents a promising model for precision medicine, given the amenability of the underlying enzymatic defects to functional characterization and therapeutic interventions. In fact, Sir Archibald Garrod, with his first description of Alkaptonuria 115 years ago, demonstrated well before the advent of whole exome sequencing (WES) that biochemical profiling in body fluids along with detailed clinical phenotyping enables the discovery of IMDs (Garrod 1902; Perlman and Govindaraju 2016). In turn, increased knowledge of metabolic pathways enables individualized treatment and prevention. Exemplary is phenylketonuria, described by Følling in 1934 after the meticulous characterization of two intellectually disabled children with a particular urinary odor, which turned out to be caused by excessive amounts of phenylpyruvic acid resulting from phenylalanine hydroxylase deficiency (Følling 1934; Centerwall and Centerwall 2000). Based on this pathophysiological insight, it was Bickel who in 1954 first treated PKU with a diet low in the accumulating phenylalanine, and Guthrie who in 1962 introduced newborn screening (NBS) to prevent the untreated sequelae of this devastating neurodegenerative disease (Mitchell et al 2011). Thus, for IMDs, “P4 medicine”—preventive, predictive, personalized, and participatory—was already practiced in the pre-omics era (Fig. 1**)** (Hood et al 2012).

Over the past century more than 700 other such conditions have been identified (Illsinger and Das 2010). Although each disorder on its own is rare, as a group, IMDs are relatively common, with a prevalence of one in 784–2555 (Sanderson et al 2006; Applegarth et al 2000; Dionisi-Vici et al 2002). These numbers obviously depend on the definition of an IMD, and in the -omics era, this is changing quickly. In 2015, Morava et al published Quo Vadis, stating that the “classification of a disorder as an IMD requires only that impairment of specific enzymes or biochemical pathways is intrinsic to the pathomechanism. If these cellular and biological processes are blocked or insufficient, they are suspected to underlie the disease phenotype” (Morava et al 2015). Thus, a genetic condition can be termed an IMD even in the absence of a biochemical phenotype or identification by metabolic lab based test. Exemplary is polyglucosan body myopathy (*RBCK1*), which is an ubiquitination defect without detectable biomarkers in blood or urine (Nilsson et al 2013). Also, some but not all congenital disorders of glycosylation are detectable by metabolic tests. These vary in number depending on the definition used maximum if the Quo Vadis definition is applied. Furthermore, IMDs are not organelle bound as illustrated by intracellular trafficking defects, such as those disrupting copper metabolism or endocytosis (Martinelli et al 2013; Stockler et al 2014), which have been shown to affect biochemical processes and intermediary metabolism. Therefore, the number of IMDs is likely significantly higher than is currently documented.

Increasingly, in selected groups of patients with symptoms such as intellectual disability, IMDs are recognized as an important etiological group. For example, in a study by van Karnebeek et al, IMDs were identified through a systematic screening algorithm using targeted mass-spectrometry as a 1st tier screening test in up to 8% of 518 patients or approximately 1/12 (Van Karnebeek et al 2014a). Importantly, the majority of these IMDs were amenable to therapy, making their identification essential and emphasizing the importance of early diagnosis in the prevention of irreversible damage to the central nervous (CNS) and other organ systems. Even in the absence of treatment, a diagnosis is important for the patient and family, as it provides closure, information and prognostication, enables more accurate genetic counseling, identification of other family members at risk, and access to community services. Still, many IMDs may go unrecognized, either because targeted laboratory diagnostics fail to identify these rare conditions, or because the genetic basis and/or phenotypic spectrum have yet to be discovered.

**Fig. 2:**
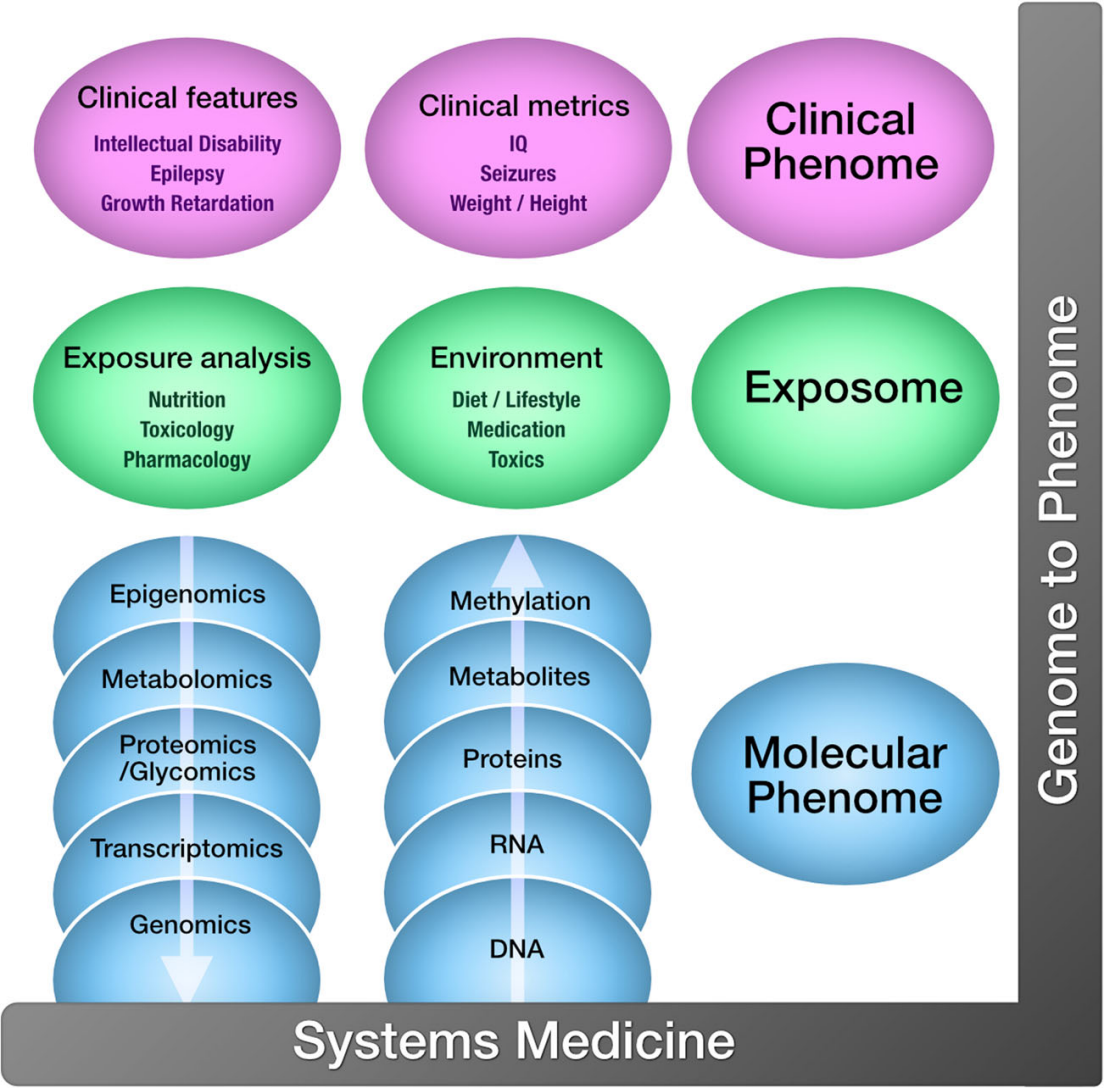
Systems biology approach to personalized medicine: Using -omics technologies to delineate the relationship between the distinct components of the clinical phenome, the exposome, and the molecular phenome, ultimately to improve pathophysiologic understanding and enhance accurate diagnostics and tailored care

The era of big data promises to accelerate the diagnostic process dramatically. Given the molecular diversity of biomarkers available, the high-throughput -omics technologies (e.g., genomics, transcriptomics, epigenomics, proteomics, glycomics, metabolomics, lipidomics), offer an amazing opportunity for holistic investigation, (Benson 2016) and contextual pathophysiologic understanding of the disease for better diagnosis and treatment (Fig. 2) (Alyas et al. 2015; Ahn et al. 2006; Tebani et al. 2016). While each of the -omics technologies is important to systems medicine, some are clearly more mature than others. WES is slowly emerging as a reimbursed test in clinics around the world, while epigenomics and “exposomics” (the environmental effects ranging from exposures to toxic substances or drugs to diet) are applied mainly in research settings. Of the MS-based technologies, metabolomics is much closer to being introduced into clinical practice than proteomics, because targeted metabolite analyses using MS have already been adopted in clinical chemistry laboratories. Whether in the research or clinical setting, all these multi-omics datasets can be generated relatively easily at low costs; the challenge, however, lies in the integration and interpretation of these systems biology data and translation of the results into something clinically meaningful for our patients. A daunting task, and this challenge can only be overcome through partnership with the patients and families and close collaboration between clinicians and researchers in the basic, computer, and clinical sciences (Julkowska et al 2017). Here we provide examples of the successful application and interpretation of high-throughput technologies, so called ‘smart-omics’, as well as the opportunities and challenges that lie ahead for precision medicine in the field of IMDs.

## Multi-omics in the clinical care for IMDs

### App-omics

The discovery of the genetic basis of IMDs and other rare diseases has accelerated with the introduction of WES; although exciting, this rapid discovery rate translates into an ever expanding group of IMDs and rare diseases the clinician must consider when evaluating a patient with symptoms of undefined etiology. Easy access to an up-to-date knowledgebase, which can be easily navigated and searched based on signs and symptoms, is essential for the clinician in the -omics era. For example, to aid in the process of comprehensive differential diagnostics and systematic screening for IMDs, metabolic protocols with tiered biochemical testing in blood, urine, and CSF have been developed (van Karnebeek et al. 2014b) as well as digitals tools to deal with the vast amount of information on IMDs such as the applications IEMbase (www.iembase.org) and Treatable ID (www.treatable-id.org) (Lee et al. 2017, 2018; van Karnebeek et al. 2012). Furthermore, the Online Mendelian Inheritance in Man (www.omim.org) is a valuable resource for all rare diseases.

### Genomics & metabolomics, targeted vs untargeted

The diagnostic yield of WES, for rare disease phenotypes is reported to be 25-30% in several large studies comprising almost 5000 rare diseases patients (Lee et al. 2014; Yang et al. 2014; Retterer et al. 2016; Wortmann et al. 2015a, b). (Taylor et al. 2015; Wenger et al. 2017) For patients with a suspected IMD based on well-characterized clinical and/or biochemical phenotypes, the WES diagnostic rate has generally been higher, ranging from 50-90% (Timal et al. 2012; Tarailo-Graovac et al. 2016; Yubero et al. 2016; Taylor et al. 2015; Evers et al. 2017; Reuter et al. 2017). With WES emerging as a highly effective front-line test for rare diseases, does metabolic testing still have a place in the diagnostic evaluation of patients with suspected IMDs, presenting with either more common symptoms such as hypotonia or epilepsy, or rare symptoms such as cerebral palsy or liver failure (De Koning et al 2015; Yohe et al 2015; Leach et al 2014)? In 2017, targeted metabolic testing still has advantages including a shorter turn-around time, which is especially important for treatable IMDs where diagnostic delay can have detrimental effects. This is best exemplified in the NBS programs, where targeted metabolic screening provides a rapid diagnosis within 3-7 days after birth allowing prevention and intervention before onset of disease (Wortmann et al 2017a, b). Second, the results overall are more straightforward to interpret than WES given the targeted nature of the tests and the availability of reference ranges. Third, metabolic testing is cheaper than WES, although if investigations are done sequentially, clearly the costs add up as does the burden for the patient and family (Genereaux et al 2015). However, methods for generating a holistic metabolic profile using one instead of multiple tests, are emerging. For example, in this issue *Coene* et al successfully identified 41 of 45 IMDs in their proof-of-principle study using a diagnostic platform based on high-resolution liquid-chromatography time-of-flight (LC-QTOF) (Coene et al. 2017). This type of broad -omics approach could potentially replace more targeted sequential metabolic testing, enhancing time- and cost-effective use of laboratory resources and providing additional data for the interpretation of the numerous variants generated by genome-wide sequencing approaches. This is especially true in the identification of a variant of unknown significance (VUS) in an actionable gene by genome-wide sequencing approach, where the diagnosis offers opportunities for treatment and mandates further investigation to confirm causality in an efficient fashion. This is where targeted and untargeted metabolic approaches can be complimentary with examples listed below.

### Phenomics still paramount

Since -omic approaches yield incidental findings as well as true diagnostic clues, careful characterization of patients along with formulation of a differential diagnosis by a clinician with broad experience in rare diseases, is more important than ever. An illustrative case vignette is a 2-year old boy who presented with progressive ataxia, neuropathy, spasticity, nystagmus, and hypomyelination. Perlizaeus-Merzbacher disease was suspected but *PLP1* molecular analysis in a CLIA certified lab was negative. The patient was enrolled in the TIDEX neurometabolic gene discovery study (University of British Columbia IRB #H12-00067). Careful inspection of this region in the trio WES data revealed a 13 bp deletion within *PLP1*. The lab improved their methods, went back to all their *PLP1* negative patients and found more patients with cryptic alterations in this region. Further, careful review of the neuro-imaging of this patient contributed to the discovery of a novel *PLP1* related phenotype termed hypomyelination of early myelinating structures (HEMS) caused by abnormal PLP1/DM20 alternative splicing due to exon 3 and intron 3 mutations (Kevelam et al 2015), further clarifying the precise relationship between gene mutation and phenotype and contributing to disease stratification. In vivo technologies such as nuclear magnetic resonance spectroscopy, radiolabeled isotype analysis to measure metabolic flux, and continuous glucose monitoring offer unique opportunities for deep phenotyping.

Meticulous characterization of patients with the same genetic defect, can improve disease understanding and identify diagnostic biomarkers (Ackerman et al 2016). Another example is RMND1 deficiency—a severe mitochondrial disease with neurologic, renal, and audiologic involvement (Janer et al 2015)—for which an in-depth neuroimaging analysis of a series of patients taught us that this disease mimics congenital CMV leuko-encephalopathy with multifocal white matter changes and temporal cysts (Ulrick et al 2017). Availability of such an imaging biomarker will facilitate diagnosis, be it with WES or more targeted testing. Another example is the “putaminal eye” seen in SERAC1 deficient patients (Wortmann et al 2015a, b).

### The functional genomics laboratory & model organisms

Even for well-known IMD, demonstrating that a novel sequence variant is pathogenic can be a challenge and ‘undoing a diagnosis’ may be even more difficult. For example in X-linked adrenoleukodystrophy, a peroxisomal IMD (*ABCD1*), leading to the accumulation of very long chain fatty acids (VLCFA), affected women suffer myeloneuropathy, while their biochemical readout VLCFA can be in the normal range. Schackmann et al reported two such women with a clinical presentation compatible with ALD, but normal VLCFA, in whom an *ABCD1* VUS was identified. Subsequent biochemical studies using clonal cell lines that express either the wild type allele or the allele carrying the VUS, showed that the two sequence variants were not pathogenic. This excluded the diagnosis of ALD in these women, who ultimately were diagnosed with multiple sclerosis, obviously with a different prognosis and treatment modalities (Schackmann et al 2016). This example illustrates the important role of the crosstalk between clinicians and laboratory specialists in coming to a diagnosis. Additional functional work is often required setting the stage for a functional genomics laboratory (Rodenburg et al 2017 (under review, this JIMD omics issue)). Model organisms including flies, yeast, worms, zebrafish, rodents, and other mammals are indispensable for the functional investigation of novel genes, variants as well as for delineation of pathophysiology and treatment strategies (Wrangler et al 2017). To accelerate rare disease discovery, the Rare Disease Model Organisms and Mechanisms was established in 2013 and demonstrates success in the connection between clinicians and model organism researchers for a large number of diseases including IMDs (Wangler et al. 2017) (Foley 2015).

### Clinical caution in the -omics era

Ultimately, the optimal approach to an undiagnosed patient with a suspected IMD remains embedded in clinical expertise and the laboratory tests available in a given jurisdiction, however, we expect these broad assessment technologies will merge in the future as a single test for a patient with a suspected IMD. At the same time, proper interpretation of these big data will require functional assays, and the clinician must be cautious in assigning etiologic diagnoses based on -omics findings alone. Mitochondrial diseases deserve special mention. Although genome-wide sequencing has greatly advanced the diagnosis of this heterogeneous group of conditions, pitfalls remain, including the need to sequence both nuclear and mitochondrial DNA (the latter extracted from affected tissue for highest levels of heteroplasmy), as well as mitochondrial ‘phenocopies’ and secondary mitochondrial dysfunction. One example is the identification of mt-ATP6 mutations as the cause of classic clinical and biochemical multiple carboxylase deficiency phenotypes, including characteristic C3 and C5OH elevations on NBS, some of whom respond to biotin (Balasubramaniam et al. 2016; Larson et al. 2018). Vice versa, examples of conditions with secondary OXPHOS dysfunction include recessive *SCN3A* mutations presenting with congenital hypotonia and respiratory complex defects (Koch et al 2017), epileptic encephalopathy, elevated alkaline phosphatase, respiratory complex deficiency in PIGA deficiency (Tarailo-Graovac et al 2015), and intellectual disability with hypotonia in TBCK deficiency (Bhoj et al 2016).

## -Omics in concert for IMD discovery

The discovery of the genetic basis of IMD and other rare diseases has accelerated over the past decade with the advent of WES. Between 2012 and 2015, international databases such as OMIM (Amberger et al 2015) and Orphanet (Rath et al. 2017) documented an average of 160 new disease-gene discoveries and 120 disease-gene relations per year (Boycott et al. 2017). It is here that the research application of genome-wide sequencing approaches and targeted and untargeted metabolic approaches are providing insight into new IMDs. For example, in a family with unexplained hyperammonemia, hyperlactatemia and hypoglycemia, a defect in intermediary metabolism close to the urea cycle was suspected but all diagnostic tests were negative. When WES data were generated, an obvious candidate gene emerged as a good fit from among the long lists of genetic variants, with the following hypothesis: it was the novel gene *CA5A* encoding carbonic anhydrase VA that had been well characterized in mice as an important enzyme involved in the production and donation of bicarbonate to four mitochondrial enzymes (carboxylases), three of which are biotin-responsive (van Karnebeek et al. 2014b). Looking back, there was also evidence of this enzymatic deficiency in the patients’ amino acids and organic acids profiles showing a multiple carboxylase deficiency phenotype, albeit subtle. The mutant enzyme was thermolabile with decreased activity at body temperature. Furthermore, the acute presentation of this IMD is amenable to carglumic acid, a synthetic N-acetyl glutamate analogue, and thus CA-VA deficiency expanded the differential diagnosis of treatable hyperammonemia in the neonate and young child (Diez-Fernandez et al 2016). In this case, elaborate pre-WES metabolic testing provided clues regarding the underlying etiology, facilitating a hypothesis driven prioritization of long variant lists generated by WES and subsequently validated by a functional analysis of the CA-VA enzyme. Another such example is the discovery of mitochondrial NADP(H) deficiency (recessive *NADK2* mutations) presenting as a fatty acid oxidation defect with hyperlysinemia and mitochondrial dysfunction due to dienoyl-CoA reductase deficiency (Houten et al 2014).

A second elegant example of this approach is the discovery of NANS deficiency in patients with intellectual disability, dysmorphisms, and skeletal dysplasia (van Karnebeek et al. 2016). WES in one of the patients, a 3-year-old boy, yielded variants in 19 different genes, whereas untargeted metabolomics performed at the same time led to the discovery of elevated levels of the metabolite N-acetylmannosamine, which could only be caused by two enzymatic deficiencies in the de novo sialic acid synthesis pathway. Putting the data sets together facilitated the disease gene (*NANS*) identification, validation of the deleterious impact of the recessive variants through accumulation of the substrate of the enzyme (N-acetylmannosamine), which also serves as a new biomarker with which multiple patients around the world have been subsequently diagnosed. Importantly, knockdown zebrafish recapitulated the human phenotype which was rescued with early sialic acid supplementation, opening up avenues for treatments similar to what is in trial for GNE myopathy, a rare muscle disease resulting from the upstream enzymatic defect (Arghov et al 2016).

## Further acceleration of IMD discovery

Despite the successes using WES approaches and functional assays to discover IMDs, at least half of patient cases remain unsolved even after detailed analysis. Reasons are myriad and likely include technical limitations (e.g., missed coding variation in WES, structural rearrangements, regulatory mutations), more complex genetic mechanisms (e.g., tissue-specific somatic mosaicism requiring biopsy of the affected organ system; two or more monogenic disorders reported in 4-9% of patients), and insufficient evidence for or against the causality of a certain candidate variant (n-of-1 challenge) (Gajecka 2016; Posey et al 2017). Next, we provide a brief overview of the possible approaches to solve the unsolved, with a focus on IMD phenotypes.

### Harnessing emerging technologies

#### Whole genome sequencing

WGS has higher sensitivity than WES for certain coding variants, indels, CNV, chromosomal rearrangements, or causative variants in regulatory regions; it is therefore postulated as an effective tool to consider in identifying unsolved IMD (Gilissen et al. 2014; Belkadi et al. 2015; Stavropoulos et al. 2016). For example, when compared to WES, WGS identifies diagnostic variants in an additional 14% of patients (deep intronic SNV, small CNV, noncoding RNA SNV) (Lionel et al 2017). Other complex rearrangements that may be detectable by WGS include inversions and transposons. The latter was reported as a cause of Salla disease in a 11-year old boy with developmental delay, thin corpus callosum, delayed myelination, and mild sialic aciduria; homozygosity for a long interspersed element-1 retrotransposon was identified in *SLC17A5* causing two new splice sites with a premature stopcodon 4 bp into intron 9 (Tarailo-Graovac et al 2017a).

#### Transcriptome sequencing

The value of transcriptome data in evaluating the functional significance of noncoding/regulatory variants in known genes was recently highlighted by several groups primarily as an adjunct diagnostic tool for the identification of mutations in known disease genes that were not identified or could not be interpreted by WES or WGS alone. Utilization of fibroblast-derived RNA identified splice mutations in known disease genes in 10% of a cohort of 48 patients with mitochondrial disease (Kremer et al 2017). Another study using RNA derived from muscle identified splice mutations in 35% of the 50 patients with undiagnosed rare muscle disorders (Cummings et al 2017). Transcriptome sequencing will also aid in identifying cases of differential allele expression resulting in (near) homologous expression of a pathogenic allele, when in an autosomal recessive disorder only one heterozygous pathogenic allele was identified by WES or WGS.

#### Epigenomics

Genome-wide methylation analysis can also identify biological signals that support disease-gene causality. For example, the methylation profiles of several rare diseases associated with genes suspected to have an impact on methylation status identified diagnostic signatures for Floating Harbor syndrome (Hood et al 2016), ATRX syndrome (Schenkel et al 2017), DNMT1-associated autosomal dominant cerebellar ataxia and deafness (Kernohan et al 2016), Sotos syndrome (Choufani et al 2015), and CHARGE and Kabuki syndromes (Butcher et al 2017).

#### Metabolomics

Although enabling ultra-sensitive, untargeted analysis of many metabolic pathways and processes all at once (amino acids and peptides, carbohydrates, cofactors and vitamins, purines and pyrimidines, fatty acids and ketones, sterols, porphyrin and heme, lysosomal, peroxisomal, lipoprotein, neurotransmission, trace elements and metals), this high mass accuracy tandem MS method is not without challenges, and it is not yet possible to analyze the complete metabolome. To start with, essential information on the effect of clinically relevant metabolite/feature information on IEM disease pathogenesis is lacking, especially as not all 10,000 features detected by the semi-automated data-processing pipelines (signals with a specific mass to charge ratio, intensity, and retention time) can be correctly annotated and reference ranges have yet to be established (Ramos et al 2017; De Sain-van der Velden et al 2017). This method, therefore, requires control samples, which are often hard to come by in pediatric populations. Further, while identification of accumulating or elevated metabolites is rather straightforward, confirming those that are decreased or ‘too low’ is complex. For metabolites that are not available as reference standard, evidence for identification is gathered from biological reference samples (patients with similar diagnosis), isotope ratios, specific in source fragmentation patterns, and available databases (such as the Human Metabolome Database (Wishart et al 2013); novel features can be further characterized using NMR-spectroscopy, infrared spectroscopy, multistage fragmentation mass spectrometry and/or targeted metabolomic analyses (Wevers et al. 1994; Martens et al. 2017; Václavík et al. 2017). As Graham et al report in a pilot study in this issue, untargeted metabolomics analyses in 15 neurometabolic patients facilitated a more holistic characterization of their phenotype, with the identification of a metabolic footprint of genetic variants with biological relevance to his/her presenting features (Graham et al. 2017). This finding is in line with the much larger study applying combined WGS and metabolomics in 1200 healthy individuals (Long et al 2017).

#### Lipidomics

In 2015, more than 100 IMDs resulting from primary or secondary defects of complex lipids synthesis and remodeling were known (Lamari et al 2015); this group of disorders, which involve many different molecules and several cellular compartments and thus not organelle-specific, is growing steadily (Saudubray et al 2015). Further expansion in this area has resulted in the ability to detect 1200 discrete and known species and >5000 (untargeted) yet non-classified lipid species/metabolites encompassing phospholipids, neutral lipids, gangliosides, sulfatides, sphingolipids, ceramides (Vaz et al. 2015, 2017).

#### Glycomics

Another quickly expanding IMD category is the field of CDGs. Early and accurate diagnosis is important since opportunities for treatment are emerging such as oral D-galactose supplementation for PGM1-CDG (Wong et al 2017). Transferrin isoelectric focusing can only identify a subset of CDGs. Therefore, *glycomic platforms* are in development that can profile O-glycans as well as N-glycans; thus, enabling the characterization of hundreds of glycoproteins and glycolipids (Van Scherpenzeel et al 2016). A further upcoming and promising technique will be the large scale analysis of glycopeptides. This will allow us to screen for the vast array of disease processes linked to the endoplasmic reticulum and Golgi organelles, many of which are yet to be characterized.

### Identification of complex genetic mechanisms

When a possible genetic diagnosis is identified by genome-wide sequencing approaches, the clinician must critically assess whether the patient’s phenotype fits previous patient descriptions. In the case of atypical symptoms and signs, the data should be re-interrogated for the presence of other variants causing other unrelated condition(s). Such blended phenotypes have been reported in 5-9% of rare disease cohorts investigated by exomes, including those with neurometabolic phenotypes (Reid et al 2016; Tarailo-Graovac et al 2016; Posey et al 2017). In addition, somatic mosaicism can cause an atypical presentation of a disease; deep sequencing is necessary for detection, ideally using DNA extracted from the affected tissue.

### Approaches to the n-of-1 challenge

The establishment of a novel gene as disease-causing requires identification of multiple unrelated patients with the same phenotype and mutations in the same gene. Multiple projects have addressed this need by developing platforms that use genotype and phenotype matching algorithms to identify cases with overlapping phenotypes and candidate genes (reviewed in Philippakis et al 2015), but were initially isolated from each other. In 2015, the international Matchmaker Exchange (MME; www.matchmakerexchange.org) was founded: a federated platform that facilitates the identification of cases with similar phenotypic and genotypic profiles (called matchmaking) through a standardized application programming interface (API) (Buske et al 2015). Seven matchmakers are currently linked via the MME connecting data from ~10,000 patients with unsolved RD. Discoveries will only grow as we connect more datasets to obtain the 50,000-250,000 unsolved cases necessary to identify the remaining RD genes as modeled using the ‘birthday paradox’ (Krawitz et al 2015). Critical to the success of such efforts are computer-readable patient descriptions; the Human Phenotype Ontology (HPO; www.human-phenotype-ontology.org) project has been developed to meet this need. Its main components are phenotype vocabulary, disease-phenotype annotations and the algorithms that operate on these. For IMDs, HPO remains underdeveloped; in this same -Omics issue Lee et al. (2018) demonstrate that a computational vocabulary comparison between IEMbase (a digital knowledge base for inherited metabolic diseases, www.iembase.org, Lee et al. 2017) and HPO revealed that only 25% of the biochemical terms in IEMbase could be mapped to HPO. The authors emphasize that contributions by the IEM clinical and research community to the curation of biochemical data are urgently needed to fully enable the application of text-based phenomics, in order to facilitate data-sharing for IMD patients.

## Personalized treatments for IMD

Precision medicine also holds the promise of individualized treatment, and challenges clinician to translate diagnosis into better management. With IMDs, ample opportunities exist for intervention once the metabolic pathways are known (Tarailo-Graovac et al 2016, Tarailo-Graovac et al 2017b). One example is pyridoxine-dependent epilepsy, which in 2005 was unraveled as a neurometabolic disease due to a lysine catabolism defect (*ALDH7A1* deficiency) with accumulation of potentially toxic metabolites (e.g., α-aminoadipic semialdehyde, α-AASA) (Mills et al 2006). Given that more than 75% of patients suffer ID despite adequate seizure control on pyridoxine (Bok et al 2012), better treatment was needed and the obvious strategy is analogous to glutaric aciduria type I, i.e., dietary lysine restriction to reduce α-AASA production and arginine supplementation to inhibit lysine transport over the blood-brain-barrier. Observational studies in small patient numbers show developmental and behavioral improvement in PDE patients on this triple therapy, especially if started early (Coughlin et al 2015). However, the evidence level to support effect of the adjunct therapy remains limited (IV), and trials are difficult to perform for reasons inherent to rare diseases such as PDE, including small patient numbers, genetic and phenotypic heterogeneity, and long-term outcomes, which are difficult to measure, and the need for standardized long term follow up to document outcome (van Karnebeek and Stockler 2012). This is a challenge for clinicians, as it requires the establishment of international collaborations and development and execution of clinical trials, often with limited time and resources available. However, it is typically the clinician who must overcome these challenges, and—especially in the absence of big pharma—drive the efforts to increase evidence and ensure optimal practice parameters and health outcomes.

Natural history studies are central to gathering evidence, while digital technologies enable us to establish patient registries that can be globally populated. With the high rate of gene discovery and treatment development in the -omics era, it is not easy for the clinician to keep track of all developments. OrphaNet sends around regular e-newsletters summarizing novel disease genes and phenotypes. In the near future the electronic patient record should theoretically enable the clinician to easily identify patients with specific unexplained symptoms seen in his/her center, so that once a disease gene is reported with a specific phenotype, individuals fitting that description can be recalled and the gene tested or the exome (re-)analyzed in a targeted fashion, which has been shown to establish diagnoses in an additional 36% of patients (Eldomery et al 2017): This endeavour is specifically relevant for and those symptoms and conditions that can be treated and symptoms, as in the two examples given here. Recessive mutations in *DNAJC12* were reported recently as a novel treatable cause of hyperphenylalaninemia, with ID and dystonia (Anikster et al 2017). Such hyperphenylalaninemia cases must have been picked up on NBS, but had remained unexplained as negative for PKU and known congenital neurotransmitter deficiencies. Given the amenability to treatment with BH4 and/or neurotransmitter precursors, these missed patients should be retro-actively identified using metabolic and genetic (re)-testing. The same holds for a recently described de novo pyrimidine synthesis disorder caused by *CAD* mutations with global developmental delay, anemia, and epileptic encephalopathy responsive to uridine supplementation (Koch et al 2017).

Secondary treatment targets can be identified by deep phenotyping combined with exome sequencing, as illustrated in an 18-year old man in whom a maternally inherited *PAK3* mutation was identified as the cause of his severe, debilitating automutilation with epilepsy, intellectual disability, and neurologic impairment. CSF neurotransmitter analysis revealed low homovanillic acid and although the pathogenesis of the dopamine deficiency was not completely understood, the mother agreed to a test of targeted intervention with Levo-Carbidopamine supplementation. Unexpectedly, the effect was sizeable; she tells the story of their diagnostic odyssey and experience with the treatment in a peer reviewed medical journal, an excerpt of which reads: “There is no cure for *PAK3* mutation or for brain damage, and Jake will continue to be at risk of a shortened life span. However, his quality of life did improve significantly once he began the medication. He is happier, less irritable. His rates of self-injury dropped dramatically. He still hits his head several times each day, but the numbers decreased quickly by 80%—from the 100 or so daily occurrences of that time. His rates have remained stable since he began the medication in August, 2015” (Bartel 2017).

Jake’s story motivates us to measure neurotransmitter metabolites in other neuropsychiatric presentations, e.g., in other epileptic patients (*SCN2A* and *SCN8A* mutations), deficiencies in CSF, and treatment with neurotransmitter precursors with clinical and biochemical improvement (Horvath et al 2016). As clinicians working closely with families, we need creativity and shared courage to tailor treatment options in the hope of alleviating symptoms. A cure it is not, but by utilizing well-designed n-of-1 trials with clearly defined outcome measures, we can certainly perform targeted interventions tailored to the individual in a safe and responsible way. Further, such cases inform us about biology and requirements for normal brain function, knowledge that might applicable not only to rare but also more common disease states.

## The future is now, actualizing the 4Ps of precision medicine for IMD

Unlike no other rare disease, IMDs are poised to actualize the 4Ps of precision medicine on a broad scale, as has been done on a smaller scale over the past century. The three major challenges we face for preventive, predictive, personalized, and participatory care for patients with IMDs are big data interpretation, translation of knowledge into clinical care, and education for application and understanding in the wider community. Inherent to big data, and its associated repositories, are the ethical and legal frameworks to utilize these datasets for discoveries that will impact patient care. The rapid advancements in data networks, storage, computation at a lower cost, and clear-cut advantages of data sharing for accurate interpretation and understanding make this seem like the critical path forward (Salerno et al 2017). However, it is challenged by data integrity, informed consent, protection of individual privacy, confidentiality, harm, data re-identification, the prevention of reporting faulty inferences and the financial investment required to maintain such infrastructure. For the busy clinician, these challenges can be overwhelming, resulting in lost opportunities: we must keep the patient at the center when it comes to data-sharing being optimally placed within day-to-day clinical workflows.

The new -omics technologies described here will all progress and at some stage be ready for clinical translation. The application of genomics in NBS programs is an active area of discussion. Theoretically, the majority of monogenic diseases, including IMD without reliable metabolic biomarker, could be identified early in life. Aside from the technical and financial challenges requiring solutions, the ethical, legal, and social implications are immense (Friedman et al 2017). Similarly, the identification of a particular genetic diagnosis may not always be predictive regarding severity of disease. Much remains to be understood regarding modifiers of disease presentation, exemplified by X-linked adrenoleukodystrophy where boys within the same family—even monozygotic twins—may develop the fatal cerebral childhood form or be largely asymptomatic. Recently included in NBS panels, this lack of geno-phenotype correlation for the 700+ reported *ABCD1* mutations poses an impediment to standardized follow-up and accurate timing of hematopoietic stem cell transplantation (Kemp et al 2016). Great care should be taken not to burden individuals that will remain asymptomatic most or all or their lives, with lifelong unnecessary medical investigations. Multi-omics should be harnessed to identify environmental and/or epi−/genomic modifiers to help prognosticate. Those accelerating or attenuating the phenotype might serve as useful treatment targets. All in all, the design of a genomic NBS roadmap weighing the interests of the different stakeholders is required.

How can we make smart -omics accessible to clinicians? Education and dissemination of knowledge on the real-life deliverables and challenges of -omics technologies for health professionals are essential to solving the current inequity. Training the next generation of clinicians to take on the role as connectors in multi-disciplinary teams is a sine qua non essential, and the latter could be structured analogous to molecular tumor boards shown effective for cancer patients. We encourage traditional metabolic laboratories to gradually shift gears, moving toward a functional genomics laboratory and begin implementing the more holistic techniques like untargeted metabolomics, lipidomics and glycomics, with the bio-informatician central to the translation of big data into personalized patient care. Open collaborations and data sharing will prevent reinvention of the wheel. Having all the right expertise at the table when discussing the diagnostic approach and individualized management plan according to the information yielded by -omics investigations (e.g., actionable mutations, novel therapeutic interventions), is the stepping stone of P4 medicine. Patient participation and the adjustment of the medical team’s plan to his/her and the family’s wishes most certainly is the capstone.

In conclusion, there is never a dull moment for the clinician in the -omics era. P4 medicine for IMDs can be achieved if we choose wisely, continuously adjust as we learn by doing, and consider the patient and family as partners central to the success of our endeavors.
